# A review of obstructive sleep apnea and lung cancer: epidemiology, pathogenesis, and therapeutic options

**DOI:** 10.3389/fimmu.2024.1374236

**Published:** 2024-03-28

**Authors:** Fang Yuan, Yanxia Hu, Fei Xu, Xujun Feng

**Affiliations:** ^1^ Department of Respiratory, The First Hospital of Jiujiang City, Jiujiang, China; ^2^ Department of Respiratory and Critical Care Medicine, The First Affiliated Hospital, Jiangxi Medical College, Nanchang University, Nanchang, China; ^3^ Department of Respiratory and Critical Care Medicine, Sleep Medicine Center, Mental Health Center, West China Hospital, Sichuan University, Chengdu, China

**Keywords:** obstructive sleep apnea, lung cancer, intermittent hypoxia, epidemiology, tumor associated macrophages

## Abstract

Despite undeniable advances in modern medicine, lung cancer still has high morbidity and mortality rates. Lung cancer is preventable and treatable, and it is important to identify new risk factors for lung cancer, especially those that can be treated or reversed. Obstructive sleep apnea (OSA) is a very common sleep-breathing disorder that is grossly underestimated in clinical practice. It can cause, exacerbate, and worsen adverse outcomes, including death and various diseases, but its relationship with lung cancer is unclear. A possible causal relationship between OSA and the onset and progression of lung cancer has been established biologically. The pathophysiological processes associated with OSA, such as sleep fragmentation, intermittent hypoxia, and increased sympathetic nervous excitation, may affect normal neuroendocrine regulation, impair immune function (especially innate and cellular immunity), and ultimately contribute to the occurrence of lung cancer, accelerate progression, and induce treatment resistance. OSA may be a contributor to but a preventable cause of the progression of lung cancer. However, whether this effect exists independently of other risk factors is unclear. Therefore, by reviewing the literature on the epidemiology, pathogenesis, and treatment of lung cancer and OSA, we hope to understand the relationships between the two and promote the interdisciplinary exchange of ideas between basic medicine, clinical medicine, respiratory medicine, sleep medicine, and oncology.

## 1 Introduction

Obstructive sleep apnea (OSA) and lung cancer are common conditions that pose serious public health risks and contribute to healthcare pressures and economic burdens on society. OSA is characterized by recurrent collapses of the upper airway (complete or partial) occurring during sleep, accompanied by intermittent hypoxia (IH), sleep fragmentation (SF), and sympathetic hyperactivity. These conditions cause a series of adverse outcomes related to cardio-metabolic and cerebrovascular diseases, among others, that seriously affect patients’ quality of life while also increasing the risk of mortality (1, 2). The current prevalence of OSA ranges from 9–38%, depending on the demographic characteristics of the investigated population (e.g., sex, age, and ethnicity); approximately 900 million adults (30–69 years old) worldwide are suffering from OSA (1, 3). The overall incidence of lung cancer has shown a certain downward trend in recent years. In contrast, concurrently, the progression-free survival (PFS) and overall survival (OS) of patients with lung cancer have been prolonged because of the undeniable recent advances in the field of oncology (4). However, lung cancer remains one of the deadliest cancers, responsible for nearly 2 million deaths worldwide in 2020, which is approximately 18% of all cancer deaths (5). Therefore, efforts to identify new risk factors for lung cancer, especially those that can be reversed with treatment, are of great importance to its prevention and therapy. Recent epidemiological studies have confirmed the increased risk of lung cancer in patients with OSA during long-term follow-up (6). Patients with lung cancer also have a higher prevalence of OSA than the normal population (7). In addition, severe OSA leads to a significantly increased risk of mortality in patients with intermediate- and advanced-stage lung cancer (8).

Given the close pathophysiologic link between OSA and lung cancer, recent preclinical studies addressing the association between the two could explain the above phenomenon. The IH and SF accompanying OSA lead to enhanced oxidative stress and chronic inflammatory responses, immune dysfunction, and loss of homeostasis in the body (9). The various pathophysiological pathways mentioned above are important contributors that may lead to the development and progression of lung carcinoma and its resistance to therapy (10). Meanwhile, OSA shares some of the same risk factors as lung cancer, such as smoking, aging, obesity, and respiratory disorders, such as chronic obstructive pulmonary disease (1, 11). These risk factors may also be significant in lung cancer progression.

There are many inconsistencies in current research, and the research fields of OSA and lung cancer are full of opportunities and challenges. Therefore, reviewing the link between OSA and lung cancer is important. First, the current epidemiological status of lung cancer and OSA comorbidity was summarized through an examination of clinical studies. Subsequently, by reviewing studies of lung cancer combined with OSA on both the cellular and molecular levels, we were able to gain a more comprehensive understanding of the pathophysiologic mechanisms between the two. Additionally, we outline the current status of therapeutics for lung cancer with OSA. Finally, we provide our insights to rationally construct future clinical diagnosis and management strategies, clinical studies, and basic research designs for patients with lung cancer combined with OSA based on the findings and limitations of current research.

## 2 Epidemiology

### 2.1 OSA in patients with lung cancer

The prevalence of OSA is relatively high among people with hypertension (12, 13), type 2 diabetes (14), hyperlipidemia (14), COPD (15), and cognitive decline (12, 16–18). This high prevalence has also been found in patients with lung cancer. Cabezas et al. evaluated 60 patients diagnosed with lung cancer using Home Sleep Apnea Testing and showed that the median apnea-hypopnea index (AHI) was 15.2 (6.4–31.2), the percentage of time spent with saturation less than 90% of the total sleep time (T90%) was a median of 11%, and 50% of patients suffered from moderate-to-severe (AHI ≥15/h) OSA (7). Similar to this study, Bhaisare et al. used polysomnography (PSG) to assess sleep in 30 patients newly diagnosed with lung cancer (29 non-small cell lung cancers [NSCLCs] and 1 small cell lung cancer [SCLC]), and the patients had a mean AHI of 12.01 ± 15.52 events/h and a mean minimum oxygen saturation of 84.53%. Patients that met the diagnostic criteria for OSA were 17 (57%), with 26.6% experiencing mild and 29.9% moderate-severe OSA (19). Another clinical cohort study from Asia confirmed that the prevalence of OSA in patients with lung cancer was 57%, with moderate-severe OSA accounting for 27% of the total, especially in patients with SCLC, an elevated AHI, and oxygen desaturation index (ODI), indicating that hypoxia may be more severe (20). Liu et al. retrospectively analyzed the data of 410 patients with newly diagnosed lung cancer, and a total of 128 patients (31.2%) had OSA, a slightly lower prevalence compared to that reported by other studies (21).

The conclusions are relatively consistent from the information provided in the few available studies. In comparison to the general population, patients with lung cancer are more likely to have coexisting OSA. The diagnosis of OSA may be overlooked in the lung cancer population. Whether this neglect has clinically significant consequences relating to prognosis will be analyzed.

### 2.2 Lung cancer in patients with OSA

OSA has been demonstrated to increase the incidence of cancer, particularly prostate, breast, and kidney cancers (22). Justeau et al. demonstrated that T90%, a parameter that measures the degree of hypoxia in OSA but not its severity, increased the incidence of cancer by approximately 30% (T90% ≥13 vs. <0.01, hazard ratio [HR] 1.33, 95% confidence interval [CI] 1.05–1.68) (23). This finding was recently confirmed by another study in which nocturnal hypoxemia associated with OSA was found to be an independent risk factor for increased cancer prevalence after fully adjusting for demographic confounders, such as age, sex, and body mass index (BMI) (24).

Simultaneously, scholars have conducted studies on whether OSA has an impact on the incidence of lung cancer. In one of the earliest studies, Gozal et al. demonstrated that OSA did not cause a statistically significant increase in the risk of developing lung cancer (HR 1.02, 95% CI 0.99–1.06) and did not correlate with the progression of lung cancer (25). Subsequently, following veterans for 7 years, Jara et al. demonstrated that OSA increased the incidence of lung cancer by 32% (HR 1.32, 95% CI 1.27–1.38) (26). Similar findings were obtained in another prospective study conducted during the same period (HR 1.52, 95% CI 1.07–2.17). When only non-smokers were included, the incidence of lung cancer in patients with OSA was approximately 3-fold that in patients with non-OSA (HR 2.96, 95% CI 1.42–6.18). However, it should be noted that for this study, an OSA diagnosis was obtained by questionnaire report rather than sleep monitoring (27). Kendzerska et al. also confirmed that severe, but not mild-moderate, OSA contributes to risk factors for lung carcinogenesis (HR 1.34, 95% CI 1.00–1.80). They found that indicators of hypoxia associated with OSA, AHI ≥28, and mean SaO_2_ <93.4% were also independent risk factors for lung cancer incidence (28). In a recent meta-analysis (comprising a total of 4,885,518 patients) pooling the above four studies on lung cancer incidence, OSA increased the risk of lung carcinogenesis by 25% (HR 1.25, 95% CI 1.02–1.53), and a further increase was noted if the follow-up time was specified to be at least 5 years or more (HR 1.32, 95% CI 1.27–1.53) (6). Another meta-analysis that included 12 studies (22) showed that, although the prevalence of lung cancer in the general population (0.023%) is slightly lower than the prevalence of prostate cancer (1.1%), OSA prevalence in lung cancer was higher (0.5%) (29). Seijo et al. demonstrated that the severity of OSA (mild, moderate, or severe) was unrelated to the prevalence of lung cancer after adjusting for age and sex. Instead, sleep parameters such as AHI (odds ratio [OR] 1.382, 95% CI 1.015–1.882) and T90% (odds ratio (OR) 1.467, 95% CI 1.121–1.920) were independent risk factors for the development of lung cancer (30). Similarly, a study by Justeau et al. confirmed that neither mild-moderate nor severe OSA increased the incidence of lung cancer, yet T90%, a parameter reflecting the degree of hypoxia, acted as an independent risk factor contributing to the development of lung carcinoma (T90% ≥13 vs. T90% <0.01; HR 2.14, 95% CI 1.01–4.54) (23).

Another clinical cohort study from China could not replicate these studies. Xiong et al. found no significant association between OSA, AHI, T90%, and the incidence of lung carcinoma in 3,786 hospitalized patients with OSA who were followed up for an average of 9 years (31). In 2019, Brenner et al. concluded a study comprising 5,243 patients with suspected OSA (mean age: 51 ± 13.1 years) with up to 5.9 years of follow-up. During this period, altogether, 265 patients were diagnosed with malignant tumors, including 14 with lung cancer (incidence rate of approximately 0.045%), and this study also failed to confirm a link between OSA and the incidence of lung carcinoma (32). Similarly, in a recently completed study, Marriott et al. demonstrated that neither AHI, a parameter reflecting the severity of OSA, nor T90%, a parameter capturing the magnitude of nocturnal intermittent hypoxia, was associated with lung cancer incidence (AHI >30 vs. AHI <5, HR 0.80, 95% CI 0.51–1.26; T90% ≥2.2 vs. T90% <0.1, HR 0.96, 95% CI 0.67–1.38) (24).

Contrary to the findings of the above studies, OSA appeared to be a protective factor for lung carcinogenesis (HR 0.87, 95% CI 0.82–0.93), as confirmed in another long-term follow-up of up to 6 years in an Asian population. In particular, for male patients, OSA was revealed to be a protective factor against the development of lung cancer (HR 0.84, 95% CI 0.78–0.90) (33). Interestingly, similar findings were obtained by Sillah et al. They anticipated 175 cases of lung cancer to occur in 34,402 patients with OSA by age–sex standardized cancer incidence ratios (SIRs); however, only 115 patients eventually presented with lung cancer (SIR 0.66, 95% CI 0.54–0.79) (34).

Overall, 7 of the 13 studies mentioned above confirmed that OSA may increase the incidence of lung cancer, including two meta-analyses with a high level of evidence; two studies found a negative association. However, we cannot conclude these studies based on their heterogeneity, including differences in study design, follow-up time, inclusion population, and reference indicators. The first aspect to consider is the variability between study designs. All but one study (30) had relatively large sample sizes; however, only one was a prospective study (27), and the diagnosis of OSA was not exclusively confirmed by sleep monitoring, even though Huang et al. examined PSG data in a sample of 108 individuals, 98% of whom met the criteria for the diagnosis of OSA. These inconsistencies can lead to a decrease in the reliability of the conclusions of the studies.

Meanwhile, among the studies, the duration of follow-up ranged from 1.9 to 9.1 years. OSA is a chronic disease, and its effects on human health can be slow and long-term (35, 36); an insufficient length of follow-up results in a reduced number of positive events, thus affecting the accuracy of the final results. In those studies that have demonstrated that OSA can raise the risk of lung carcinogenesis (26–28), the average duration of follow-up was more than 7 years. The second drawback is that although the studies, including two meta-analyses, have demonstrated that OSA increases the risk of lung cancer, only Seijo et al. (30) and Kendzerska et al. (28) have demonstrated a causal relationship between OSA-associated hypoxia (AHI, ODI, mean SaO_2_) and the elevated incidence of lung cancer. It is also noteworthy that mean SaO_2_ is more reflective of the degree of nocturnal hypoxia rather than being a marker specific to IH. Considering that many confounding factors accompanying OSA, such as aging, smoking, and obesity, also increase the risk of lung cancer, it would be highly arbitrary to generalize that IH because of OSA increases the incidence of lung cancer. Finally, these studies did not consider a pathophysiological feature of OSA, namely SF. The duration of sleep is linked to the progression of cancer, and sleep deprivation increases the risk of its development (37, 38). For OSA, SF is an objective reflection of sleep deprivation, and SF interacts with IH to create a vicious cycle. For example, IH can lead to microarousals and, therefore, to SF. In turn, decreased arousal thresholds and SF lead to apneic events and IH (39). Pathophysiologically, SF is still distinct from sleep deprivation and shortened sleep duration alone. There is a lack of consistent metrics to quantify SF, which may limit studies on the correlation between SF and lung cancer. However, it would be interesting if multiple parameters in PSG that reflect arousal and SF (e.g., microarousal index, electroencephalogram (EEG)-based sleep staging conversion, and electroencephalographic energy changes) were to be included in future studies.

### 2.3 Prognosis of coexisting OSA and lung cancer

OSA has a high prevalence among patients with lung cancer. AHI >30/h has been shown to result in a higher risk of all-cause mortality (40), while lung cancer is also a common cause of death (41); however, it is unclear whether the combination of the two would further increase the risk of mortality. Prognostic studies addressing concurrent OSA and lung cancer are limited. The results of a follow-up study of 45 patients with lung cancer (24 combined with OSA) confirmed that in the OSA group, a total of 10 deaths and 9 recurrences or metastases occurred, with a significantly greater overall rate of deterioration (death + recurrence or metastasis) than that in the non-OSA subgroup (5 deaths and 5 recurrences or metastases) (42). Liu et al. conducted a study of 44 patients with NSCLC (OSA: 22, non-OSA: 22) with a 2-year follow-up. The survival rates of patients with OSA were lower than those without sleep apnea (1-year: 90.9% vs. 95.5%, 2-year: 65.8% vs. 71.6%), although no significant difference was found between the two groups based on the log-rank test. Vascular endothelial growth factor (VEGF) is a proangiogenic cytokine that contributes to the development of solid tumors by promoting tumor angiogenesis (43). Currently, various anti-VEGF drugs, such as bevacizumab, are being utilized for tumor treatment. After measuring the plasma levels of VEGF, the study found that the expression of VEGF was significantly higher in the OSA+NSCLC group (44). A study by Huang et al. confirmed that in intermediate and advanced lung cancer (stages III and IV), severe OSA increased the risk of death, with 80% of patients with severe OSA+ lung cancer dying within three years. Patients with mild-moderate OSA had longer PFS and OS than the severe OSA group (8). Data from the above three studies were included in a recent meta-analysis (a total of 112 patients with lung cancer, including 67 with coexistent OSA), and it appeared that OSA did not increase the risk of death from lung cancer (OR 2.005, 95% CI 0.703–5.715) (37).

To date, no studies have confirmed an increased risk of mortality from lung cancer caused by OSA. However, drawing such a conclusion might be hasty. It could even misdirect the clinical management of this group of patients, given the high prevalence of OSA in patients with lung cancer. Our rationale is as follows. First, the studies included a very small population, with sample sizes totaling less than 200. Second, factors associated with the prognosis of lung cancer include clinical stage and pathologic and molecular type, which are elements that need to be clarified in the clinical management of lung cancer. Third, there are many indicators for evaluating the prognosis of lung carcinoma, such as disease-free survival (DFS), PFS, OS, and 3- or 5-year survival rates (45, 46). Study designs should not be one-sided based on a single outcome metric. Finally, among the above studies, except for one with a follow-up duration of approximately 5 years (8), the median follow-up time in the other two was less than 2 years (42, 44), and the shorter follow-up time could affect the reliability of the study conclusions. By including a large sample of patients with lung cancer, stratifying the design according to lung cancer type, referring to several outcome indicators, and using the last patient death as the follow-up endpoint, the conclusions of the studies can be made more convincing. Unfortunately, none of the current studies have addressed these issues. Accordingly, we cannot yet conclude this issue, i.e., whether OSA affects the prognosis of patients with lung cancer.

### 2.4 Summary

Epidemiologic studies addressing the correlation between lung cancer and OSA have reported mixed results (**Table 1**). Limited findings indicate that OSA is relatively common in patients with lung cancer. OSA is also associated with an increased incidence of lung cancer. To date, there is a lack of direct evidence that OSA raises the mortality of lung cancer. Based on the above analysis of the study limitations, we believe that there is an urgent need to further improve the quality of studies, e.g., by conducting prospective, large-sample, multicenter studies and setting the lung carcinoma incidence and prognosis to the primary outcomes, to draw more convincing conclusions and guide clinical practice.

**Table 1:** Summary of the epidemiology of OSA and lung cancer.

Author, year	Design	Sample sizes	Age (years)	Male (%)	Types of lung cancer	OSA diagnosis	Follow-up duration (years)	Adjustments	Main findings	References
OSA in patients with lung cancer										
Cabezas, 2018	NR	60	67.8	58	NSCLC (83.3%) SCLC (16.7%)	HSAT	NR	NA	48/60, 18 (30%) with mild, 30 (50%) with moderate-to-severe SDB	(7)
Liu, 2022	Retrospective clinical cohort	410	59.98	58	NSCLC (82.2%) SCLC (17.8%)	Portable sleep recorder	NR	NA	128/410	(21)
Lee, 2022	NR	69	68	73	NSCLC (88.4%) SCLC (11.6%)	HSAT	NR	NA	39/69, 21 (30%) with mild, 10 (15%) with moderate, and 8 (12%) with severe SDB	(20)
Bhaisare, 2022	Prospective clinical study	30	55	87	NSCLC (97%) SCLC (3%)	PSG	NR	NA	17/30, mild, moderate, and severe OSA were seen in 26.6%, 16.6%, and 13.3%	(19)
Lung cancer in patients with OSA: incidence or prevalence										
Gozal, 2016	Retrospective matched cohort	3408906	50–59, range	50.2	NR	ICD-9-CM	1.87–3.91, range	Age, sex, morbid obesity, hypertension, type 2 diabetes, ischemic heart disease, coronary heart failure, stroke, cardiac arrhythmias, and depression	HR: 1.09, CI: 1.05–1.13; HR: 1.02, CI: 0.99–1.13 (After adjustment)	(25)
Jara, 2020	Retrospective matched cohort	1377285	55.2	94	NR	ICD-9	7.4	Age, sex, year of cohort entry, race, smoking status, alcohol use, obesity, and comorbidity	HR: 1.32, CI: 1.27–1.38	(26)
Huang, 2021	Prospective cohort	65330	73.3	0	NR	Self-reported. 108 people were sampled for PSG, OSA=98%	8	Age, ethnicity, family history of cancer, BMI, height, pack-years of smoking, alcohol drinking, physical activity, sleep duration, duration of hormonal therapy use by type, history of type 2 diabetes, aspirin use, and recent physical examination	HR: 1.52, CI: 1.07–2.17; HR: 2.96, CI: 1.42–6.18 (in never-smoked OSA patients)	(27)
Kendzerska, 2021	Retrospective cohort	33997	50	58	NR	PSG	7	Sleep clinic site, age, sex, alcohol use disorder, prior CHF, COPD, hypertension, diabetes, and OSA treatment	Severe OSA vs. Non-OSA: HR: 1.38, CI: 0.94–2.04; Q4 (AHI ≥28) vs. Q1 (AHI <4): HR: 1.78, CI: 1.03–3.10; Mean SaO_2_: Q4 (SaO_2_ ≥96%) vs. Q1 (SaO_2_ <93.4%): HR: 2.05, CI: 1.01–4.15	(28)
Justeau, 2020	Retrospective cohort	8748	61	64.50	NR	PSG	5.8	Age, sex, BMI, smoking status, alcohol intake, diabetes, hypertension, medical history of cardiac disease and COPD, and marital status	T90% (percent nighttime with oxygen saturation <90%) ≥13 vs. T90% <0.01, HR: 2.14, CI: 1.01–4.54	(23)
Brenner, 2018	Retrospective cohort	5243	51	74.50	NR	PSG	5.9	Age, sex, and BMI	OSA does not increase the incidence of lung cancer	(32)
Marriott, 2023	Retrospective cohort	20289	NR	68.70	NR	PSG	11.2	Age, BMI, sex, and smoking status	HR: 0.80, CI: 0.51–1.26; T90% ≥2.2 vs. T90% <0.1, HR: 0.96, CI: 0.67–1.38	(24)
Sillah, 2018	Retrospective cohort	34402	51.6	54.70	NR	ICD	5.3	Age and sex	Age–sex standardized cancer incidence ratios (SIR): 0.66, CI: 0.54–0.79	(34)
Park, 2023	Retrospective cohort	1607094	45.7	75.80	NR	ICD-10	5.9	Sex, age, subjects’ income levels, diabetes, hypertension, dyslipidemia, stroke, chronic obstructive pulmonary disease, and ischemic heart disease	HR: 0.87, CI: 0.82–0.93; Male OSA, HR: 0.84, CI: 0.78–0.90; Female OSA, HR: 1.05, CI: 0.91–1.21;	(33)
Seijo, 2019	Cross-sectional study	302	64.7	58.60	NR	HSAT	NR	Age, sex, smoking, alcohol consumption, emphysema, BMI, neck circumference, basal oxygen saturation by pulse oximetry and sedative consumption	AHI (ln), OR: 1.382, CI: 1.015–1.882; ODI 3% (ln), OR: 1.452, CI: 1.056–1.997;	(30)
Xiong, 2022	Retrospective cohort	4623	62.6	80.70	PSG	PSG	9.1	Sex, age, BMI, smoking status, hypertension, CHD, type 2 diabetes, and hyperuricemia	OSA does not increase the prevalence of lung cancer	(31)
Lung cancer in patients with OSA: prognosis										
Liu, 2020	NR	44	NSCLC:63.55; OSA+ NSCLC:58.45	63.60	NSCLC	PSG	1.8	TNM stage III-IV; Refusal of treatment; Pretreatment TGF-β1 level	Pretreatment VEGF (level >950 pg/ml), HR: 1.003, CI: 1.001–1.005 (multivariate); OSA, HR: 1.06, CI: 0.36–3.16 (univariate);	(44)
Liu, 2019	NR	45	NR	NR	NR	Portable sleep recorder	1	NR	The overall rate of deterioration (death + recurrence or metastasis) was significantly higher in the OSA group than in the non-OSA subgroup (P<0.05)	(42)
Huang, 2020	Retrospective	16	62.4	94%	III and IV lung cancer	PSG	5	AHI, T90%, and Eastern Cooperative Oncology Group status	Kaplan–Meier survival analysis, patients with stage III–IV lung cancer and AHI <30 events/h exhibited significantly better overall survival (P=0.02) and progression-free survival (P=0.02) than patients with severe OSA	(8)

AHI, apnea-hypopnea index; BMI, body mass index; CHD, coronary heart disease; CHF, chronic heart failure; COPD, chronic obstructive pulmonary disease; HSAT, home sleep apnea test; ICD, International Classification of Diseases; NA, not available; NR, not reported; NSCLC, non-small cell lung cancer; PSG, full polysomnography; SCLC, small cell lung cancer; SDB, sleep-disordered breathing; T90%, percent night time with oxygen saturation <90%; TGF, transforming growth factor.

## 3 Pathogenesis

What role do IH and SF, linked to OSA, play in the origin and progression of lung cancer? Research on this topic has progressed from macro to micro, from cellular to molecular, from the initial study of the size and weight of tumor tissues to the further study of the tumor-associated microenvironment and the genetic material within tumor cells. Early studies of the pathophysiologic link between OSA and lung cancer included how to construct animal models of IH that mimic similarities to OSA and explore the impact of IH on the progression of lung cancer. Lim et al. successfully constructed a mouse model mimicking OSA+NSCLC by injecting adenovirus into Kras^G12D+^, p53^fl/fl^ genetically engineered mice and stimulating them with hypoxemia. They found that IH increased the volume of primary lung tumors (P<0.001), which also increased significantly faster than that of the control group (47). Huang et al. used 7-week-old male C57BL/6 mice and Lewis lung carcinoma cells that were stimulated with nitrogen and oxygen to simulate the IH of OSA (120 s per cycle, minimum oxygen concentration of 6% for 8 h for 5 weeks) and successfully constructed an OSA+NSCLC mouse model. This study confirmed that programmed cell death-ligand 1 (PD-L1) expression in lung cancer tissues was positively correlated with IH (r=0.911, P=0.015). Compared with the NSCLC group, the weight and volume of lung cancer tissues of the OSA+NSCLC group were substantially increased. It also confirmed that the increase in weight and volume of tumor tissue showed a dose-dependent relationship with PD-L1 expression (P<0.05) (48).

### 3.1 Tumor immune microenvironment

The tumor immune microenvironment (TIME) comprises immunosuppressive molecules, matrix components, inhibitory immune cells, and cytokines. Its primary function is attenuating the anti-tumor immune response, sustaining cell proliferation, preventing cell apoptosis, maintaining an immunosuppressive milieu, and facilitating angiogenesis (49, 50). Xie et al. (51) discovered that the responses of various immune cell types in the peripheral blood of patients with OSA exhibited dissimilarities and were positively associated with the severity of hypoxemia. Studies have demonstrated that IH exerts its immunosuppressive effects through various hypoxia-inducible factor-1a (HIF-1α)-related signaling pathways: inducing remodeling of the HIF-1α metabolic pathway and enrichment of lactate in the TIME; impeding T-cell proliferation, tumor invasion, and cytokine production; and augmenting myeloid-derived suppressor cell (MDSC) numbers while inhibiting natural killer (NK) cell and CD8^+^T cell activity (52). Simultaneously, IH-induced tumor cells release interleukin 10 and promote M2-type differentiation of tumor-associated macrophages (TAMs) (53), which exhibit immunosuppressive properties. This further fosters the accumulation of other immunosuppressive populations, such as MDSCs, granulocytes, and regulatory T cells (Tregs) (54). Akbarpour et al. constructed a murine model of NSCLC *in vitro* that mimicked OSA, which demonstrated that IH and SF reduced tumor killing by cytotoxic T lymphocytes (CTLs) by significantly decreasing the secretion of granzyme B by CTLs (55). The alteration in immune function allows cancer stem cells (CSCs) to undergo immune evasion and maintain their self-renewal capacity, the growth and progression of lung cancer (55).

TGF-β plays a pivotal role in the initiation and progression of tumorigenesis. The classical and non-classical signaling pathways mediated by TGF-β participate in tumor cell growth, invasion, and metastasis and play a crucial role in regulating diverse immune cell types within the tumor microenvironment. TGF-β has emerged as a central immune regulator in the tumor microenvironment, and its combination with immunotherapy can exert significant anti-tumor effects (56). An analysis of the phenotype and immune response activity of 29 healthy volunteers and 32 patients with continuous positive airway pressure (CPAP)-treated OSA revealed that glycoprotein-A repetitions predominant protein monocytes from untreated patients with OSA inhibit NK cells through the release of TGF-β; however, reoxygenation eventually restores their altered phenotype and cytotoxicity (57). Subsequent studies have demonstrated that IH can enhance lung cancer cell migration by upregulating the TGF-β signal, increasing the activation and proportion of lung cancer-associated fibroblasts (CAFs), thereby promoting lung cancer progression (58).

The programmed cell death protein 1/programmed cell death-ligand 1(PD-1/PD-L1) pathway is an important signaling pathway for the body to negatively feedback suppress immune responses and maintain autoimmune homeostasis (59, 60). In the tumor microenvironment, the over-activated PD-1/PD-L1 pathway inhibits the body’s immune surveillance and immune clearance effects on tumor cells (61). In a murine model of sleep apnea (48), PD-L1 expression levels were significantly increased in the intermittent hypoxia group, and HIF-1α levels were strongly correlated with PD-L1 expression. In a multicenter observational study, 360 patients with cutaneous melanoma were enrolled, and serum soluble programmed death ligand 1 (sPD-L1) levels were measured, which showed that sPD-L1 levels were higher in patients with severe OSA than in those with non-severe OSA and those with non-OSA (62). Carolina et al. showed that IH-induced PD-L1/PD-1 overexpression in patients with severe OSA reduced autologous T cell proliferation and cytotoxic activity of CD8^+^T cells, increased recruitment of myeloid-derived suppressor cells, and showed an increase in PD-L1 on monocytes and PD-1 on CD8^+^T cells in patients with OSA (63). Theoretically, the higher the PD-L1 expression, the stronger the tumor cell immunosuppression. IH associated with OSA promotes the expression of PD-L1 in tumor cells, and by blocking the binding between PD-1 and PD-L1, PD-1/PD-L1 inhibitors enable immune cells to maintain their activity and restore their recognition and killing of tumor cells. Therefore, we speculate that PD-1/PD-L1 inhibitors may have a significant effect on the treatment of lung cancer patients with OSA, which might be an interesting area of research to be further confirmed in the future.

### 3.2 Tumor-associated macrophages

TAMs, MDSCs, and Tregs are involved in tumorigenesis, invasion, and metastasis. TAMs can be differentiated from MDSCs and are involved in constructing tumor stroma. MDSCs themselves can suppress T cell responses. Under various chemokines and cytokines, monocytes are recruited to the periphery of lung cancer cells and differentiate into TAMs. Under hypoxic conditions, TAMs secrete mitogenic factors and immunosuppressive agents that promote the progression of lung cancer (64). The phenotypes that make up human TAMs include two main types, M1 and M2. M1-TAMs play a role in inhibiting lung cancer progression by killing tumor cells.

**Figure 1:**
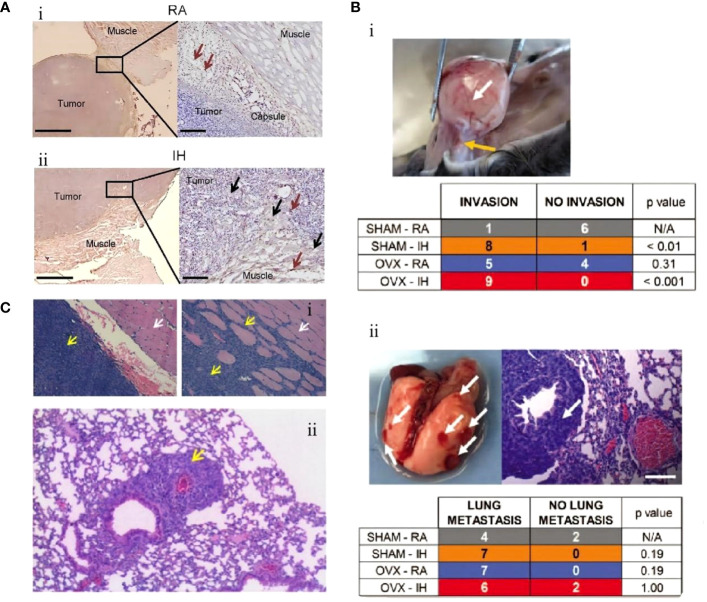
Intermittent hypoxia (IH) facilitates lung cancer progression by mediating tumor-associated macrophages (TAMs). **(A)** Comparison of TAMs in room air (RA) and IH groups. (i) In the RA group, TAMs are present in the connective tissue surrounding the tumor (brown arrow). (ii) In the IH group, in the IH state, the invasion of lung cancer is enhanced, and TAMs are present in the connective tissue (brown arrow) and muscle (black arrow) surrounding the tumor (67). **(B)** Effects of IH and oophorectomy (OVX) on invasion and distant metastasis of lung cancer in mice. (i) In IH, the primary lung cancer (white arrow) infiltrates the skeletal muscle (yellow arrow). (ii) When exposed to both IH and OVX, lung cancer metastases (white arrows) increased (68). **(C)** The invasive and metastatic potential of lung cancer in IH and RA are compared. (i) RA group (left), no invasion; IH group (right side), lung cancer invading muscle tissue. Cancer cells (yellow arrows), muscle cells (white arrows). (ii) IH stimulates lung cancer metastasis, and the yellow arrow indicates metastatic lesions (69). Reprinted with permission from Ref (67–69).

Conversely, M2-TAMs are promoters for tumorigenesis and progression. They inhibit cellular immune responses and the activity of natural killer cells (NK cells), releasing inflammatory factors and protein hydrolases. They are considered important immunosuppressive cells in the tumor microenvironment (65). The polarization process (M1 to M2) of TAMs, i.e., the decrease in M1/M2 values, has also been proposed to predict the poor prognosis of lung cancer (66). IH associated with OSA can further promote the growth of lung cancer tissues by promoting the differentiation and polarization of TAMs (M1 to M2), as well as increasing the invasiveness to the surrounding tissues and distant metastasis of lung cancer cells. Almendros et al. constructed an IH model mimicking OSA and implanted a lung epithelial tumor into mice. They found lung carcinoma cells in the IH group were more invasive than those without hypoxia after 28 days. Concurrently, compared to the control group, there was a 2.6-fold increase in TAMs around the tumor tissue (P=0.02), with a simultaneous increase in Tregs and MDSCs (2.2-fold and 3.7-fold, respectively). Applying TAMs isolated from IH mice to *in vitro* cultures significantly increased tumor cell proliferation, migration, and invasiveness (**Figure 1A**) (67). A similar study also confirmed that IH-exposed mice had significantly higher lung cancer tissue weights (1.363 ± 0.143 g) than hypoxia-free mice (0.677 ± 0.097 g), with a 2.2-fold increase in IH-induced TAM infiltration, which further confirmed that prolonged exposure to IH induces adipose tissues to recruit adipose tissue stem cells (ASCs) and pro-inflammatory cells, leading to the migration of adipose tissue macrophages and ASCs to tumors, promoting tumor tissue growth and enhancing its invasiveness (70). The effect of sex on the relationship between IH and lung cancer was first explored by Torres et al. by mimicking postmenopausal mice (oophorectomized). They demonstrated that, compared to mice exposed to CH, IH promoted greater lung cancer tissue growth in ovariectomized mice. Although not statistically significant (P=0.08), this study also demonstrated that IH promotes TAM aggregation around tumor tissues and facilitates the conversion of TAM phenotype from M1 to M2 (**Figure 1B**). This study confirmed that IH associated with OSA can facilitate the progression of lung carcinoma (68). Recently, a study confirmed that TAMs and PD-L1 expression were upregulated in patients with OSA with lung adenocarcinoma. OSA-related IH activated HIF-1α and its associated signaling pathways, enhanced the expression of PD-L1 in tumor cells, and increased the activity of TAMs while decreasing cytotoxic T cell activity. Lung cancer growth was promoted through the tumor-associated immune response mediated by IH (71). Campillo et al. constructed an IH+NSCLC model *in vivo* and *in vitro* (69). The exposure of IH promoted the proliferation of lung cancer by upregulating the number of TAMs as well as mediating the polarization of the phenotype of TAMs (the ratio of M2/M1 was increased 2-fold in the IH group compared to that in the control group). Cyclooxygenase-2 and its downstream secreted prostaglandin E2 (PGE2) were shown to be important mediators in regulating this process. PGE2 expression was 1.5-fold higher in the IH+NSCLC group (**Figure 1C**). This study confirmed that OSA-associated IH exacerbated lung cancer progression by activating the inflammatory response pathway (COX-2/PGE2) and promoting the proliferation of TAMs (69).

### 3.3 Cancer stem cells

Since the proposal of CSCs by Mackillop et al. in 1983 (72), they have been acknowledged as a highly tumorigenic subpopulation capable of facilitating tumor growth, drug resistance, recurrence, and metastasis through the process of epithelial-mesenchymal transition (EMT) reprogramming (73, 74).

**Figure 2:**
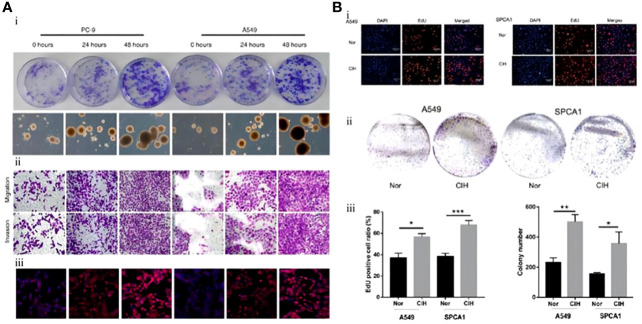
Mouse lung cancer cells exposed to intermittent hypoxia (IH) had greater stem cell potential and stronger proliferation and differentiation ability. **(A)** Lung cancer cell lines (PC-9, A549) were exposed to IH. (i) Colony formation of lung cancer cell lines. CSCs measured at 0 h, 24 h, and 48 h after exposure to IH. (ii) Immunohistochemistry of CSCs (PC-9, A549). (iii) Immunofluorescence staining of CSCs (PC-9, A549) (75). **(B)** Lung carcinoma cell lines (A549, SPCA1) were exposed to IH. (i) After exposure of A549 or SPCA1 to normal oxygen (Nor) or chronic intermittent hypoxia (CIH) for 48 h, cell proliferation was assessed with EDU and DAPI staining. (ii) Colony formation analysis shows that IH could promote the proliferation of CSCs (A549, SPCA1). (iii) Comparison of EDU staining positive ratio in CSCs (A549, SPCA1) between Nor and CIH (left) and comparison of CSC (A549, SPCA1) colonies between Nor and CIH (right) (76). Reprinted with permission from Ref (75, 76).

The study by Gu et al. conclusion that IH promotes the differentiation, proliferation, invasion, and migration of CSCs through HIF-1α and can simultaneously induce drug resistance. This study also identified another important molecule: endothelial cell-specific molecule-1 (ESM1), and using an ESM1 antagonist, they reversed the negative effects of IH on a murine model of NSCLC (**Figure 2A**) (75). *In vitro* and *vivo*, Hao et al. demonstrated that IH promoted tumor proliferation, invasion, and distant metastasis and identified a series of mechanisms by which IH promotes the progression of NSCLC through the upregulation of CSC expression. *In vivo* and *in vitro*, IH promoted the aggregation of mitochondrial reactive oxygen species (mtROS), mediated oxidative stress, and induced the generation of more CSCs by upregulating the expression of transcription factor BTB and CNC homology 1 (Bach1). This response mediated by IH disappeared after gene silencing of Bach1 (**Figure 2B**) (76). Meanwhile, by measuring the levels of HIF-1α and ATPase family AAA structural domain-containing protein 2 (ATAD2)-associated genetic products in A549, H460, H1299, and SPC-A1 cells, which are the representative NSCLC cells, the levels of ATAD2-related mRNA and protein levels were found to be significantly elevated in the IH+NSCLC model. Knockdown of ATAD2 significantly inhibited the invasion and migration of lung carcinoma cells. The production of mtROS and the number and activity of CSCs were also reduced. This study confirmed that IH regulates the interaction between mtROS and CSCs by mediating HIF-1α/ATAD2, promoting lung cancer progression (77).

### 3.4 Endothelial cells

Continuous neovascularization guarantees the tumor tissue’s thirst for nutrients, necessary to maintain lung carcinoma cells’ proliferation, infiltration, and distant metastasis. Vascular endothelial cells are the cornerstone in this process, and VEGF promotes lung cancer carcinogenesis and progression by inducing the differentiation and maturation of endothelial cells (78, 79).

**Figure 3:**
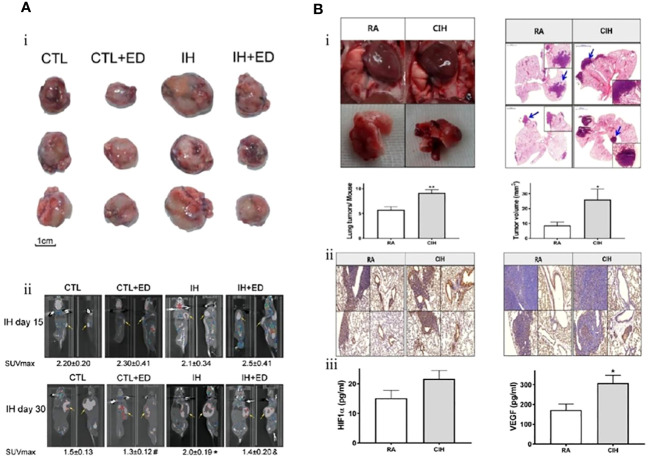
Intermittent hypoxia (IH) promotes endothelial cell proliferation through upregulation of vascular endothelial growth factor (VEGF) expression and ultimately accelerates lung cancer progression. **(A)** Comparison of tumor size in each group. (i) The tumor size of lung cancer in the IH group was larger than that in the control (CTL) group. Endostatin (ED) inhibits the proliferation of endothelial cells (CTL+ED, IH+ED) and slows tumor growth. (ii) After 15 and 30 days of exposure to IH, 18F-FDG PET demonstrated tumor size. Endothelial cells were the target of ED treatment, and lung cancer proliferation is inhibited by ED. Yellow arrows point to tumors (80). **(B)** In contrast to room air (RA), chronic intermittent hypoxia (CIH) promotes lung cancer proliferation by inducing neovascularization. (i) The number and volume of tumors are larger in the CIH group. (ii) The effect of CIH on the neovascularization of lung cancer. Representative immunohistochemistry images (left: Ki-67 and right: CD31) (magnification, ×200). (iii) Hypoxia-inducible factor-1a (HIF-1α) and VEGF expression levels in the CIH group are higher than those in the RA group (81). Reprinted with permission from Ref (80, 81).

Zhang et al. investigated the expression of serum VEGF and endothelin-1 in lung cancer associated with IH. Their results showed that the volume and weight of tumors exposed to IH were significantly higher than those of tumors under normal oxygen conditions (3974.50 ± 1748.20 vs. 2268.54 ± 1874.48 mm^2^ and 5.76 ± 1.48 vs. 4.49 ± 0.96 g, respectively). VEGF and its mRNA levels were also significantly increased in serum as well as tumor tissues of mice. This research also demonstrated that IH promoted the proliferation and differentiation of endothelial cells by upregulating the expression of VEGF and endothelin-1, which accelerated the progression of lung cancer by inducing neovascularization in tumors (**Figure 3A**) (80). The same finding was replicated in another study during the same period. Kang et al. confirmed the role of VEGF and endothelial cells in promoting IH-mediated lung cancer progression. The expression level of VEGF was markedly higher in the IH+lung adenocarcinoma mice model than in the normoxia+lung adenocarcinoma group (308.1 ± 104.3 vs. 172.0 ± 90.6 pg/mL). Nuclear factor-erythroid 2-related factor 2 (Nrf2) and Beta-catenin (β-catenin), but not HIF-1α, appear to be the key intermediary mediators promoting VEGF expression (**Figure 3B**) (81).

Serum midkine (MDK), as a lymphangiogenesis-related biomarker, its overexpression has been identified in patients with NSCLC as a potential therapeutic target for its development, metastasis, and angiogenesis (82, 83). Lymphangiogenesis, in addition to angiogenesis, mediates tumor vascularization, which meets the oxygen and nutrient requirements for tumor growth and metastasis (84). Recent studies have shown that individuals at high risk for lung cancer with moderate-to-severe OSA have elevated expression of MDK (non-OSA: 1536 pg/mL, 95% CI 840–2360 pg/mL; moderate-to-severe OSA: 5902 pg/mL, 95% CI 816–8337 pg/mL) (85).

### 3.5 Exosomes

Exosomes are important bioactive factors that can participate in cell communication, immune response, cell differentiation, and tumor invasion and can also be used as carriers for drug therapy (86, 87). Exosomes increase the proliferation of lung cancer by stimulating glucose anaerobic fermentation metabolism and ensuring oxygen supply to lung cancer cells by promoting neovascularization during lung cancer carcinogenesis and progression. Concurrently, exosomes can induce EMT and deliver regulatory factors between cells, thus promoting distant tumor metastasis (88).

**Figure 4:**
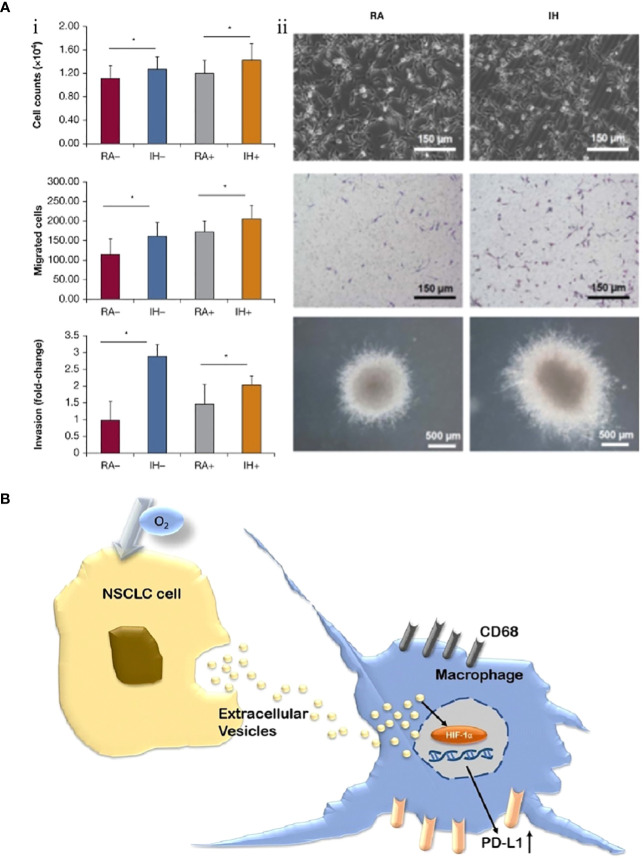
Exosomes are involved in regulating the progression of lung cancer under intermittent hypoxia (IH) conditions. **(A)** IH promotes the proliferation of lung cancer cells and is associated with exosomes. (i) Effects of exosomes derived from room air (RA) or IH mouse plasma on proliferation, migration, and invasion of mouse lung cancer cells. (ii) Comparison of lung cancer cell proliferation between RA and IH under electron microscope, light microscope, and *in vitro* colony (89). **(B)** Non-small cell lung cancer (NSCLC) cell-derived exosomes contribute to the progression of NSCLC by promoting the upregulation of PD-L1 expression in macrophages (90). Reprinted with permission from Ref (89, 90). *P<0.01.

Almendros et al. extracted exosomes from blood samples from 10 adult patients with OSA in fractions (before CPAP treatment and after 6 weeks of treatment) and confirmed that exosomes before CPAP treatment significantly enhanced the differentiation, maturation and migration of adenocarcinoma cells, which was validated in an animal model. In parallel, this study also identified several miRNA sequences (such as mmu-miR-92a-3p and mmu-miR-709) associated with exosomes (**Figure 4A**) (89). Subsequently, a study by Liu et al. also confirmed that exosomes derived from lung cancer cells under IH-exposed conditions upregulated the expression of PD-L1 on the surface of macrophages surrounding tumor tissues and promoted lung cancer progression (**Figure 4B**). Compared with patients with lung cancer without OSA, the percentage counts of monocytes positively expressing PD-L1 were markedly higher in serum samples from patients with lung carcinoma with concomitant OSA (95.3% vs. 86.0%), and this result was positively related to the parameters AHI and ODI, which reflected the severity of OSA (r=0.419 and 0.387, P<0.05, respectively) (90).

### 3.6 Genetic materials

The metabolism of normal tissues and cells results from a combination of multiple gene regulation processes. Proto-oncogenes promote the process of cell mitosis and developmental maturation by regulating the cell cycle. Oncogenes mainly prevent excessive cell proliferation. The dynamic balance between the two maintains the normal metabolism of the body. The carcinogenesis and progression of lung carcinoma is the adverse consequence of dysregulation of proto-oncogenes/oncogenes (91–94).

Genetic material is equally vital in regulating the carcinogenesis and progression of OSA-related IH and lung carcinoma. Cortese et al. studied NSCLC mice exposed to IH. They confirmed a significant increase in the amount of cirDNA compared to NSCLC mice in normoxic environments by measuring cirDNA levels in plasma (difference between the two: 510.62, P=0.015). Meanwhile, with the increase in the amount of cirDNA, the size and weight of the tumor increased (R^2 =^ 0.580 and 0.765, respectively, P<0.05). These cirDNAs may carry specific epigenetic modifications for information transfer between OSA and lung cancer (95). RNA is an important carrier of genetic information, controlling cell growth and differentiation by conducting protein synthesis, transmitting genetic information, and regulating transcription and translation (96). Chao et al. constructed human lung adenocarcinoma cell lines exposed to IH *in vitro* and inhibited the proliferation and invasiveness of IH-exposed tumor cells by knocking down the demethylation enzyme ALKB homologous protein 5 (ALKBH5). This process may be mediated by downregulation of the level of n6-methyladenosine (m6A)-modified mRNAs. This finding was confirmed *in vivo* and *in vitro* (97). MicroRNAs are a class of small RNAs about 20–24 nucleotides in length that do not encode proteins but can regulate the translation of mRNAs. Li et al. identified a specific fragment of genetic material, microRNA-320b. In patients with lung cancer who also experienced OSA, IH induced a decrease in the expression of microRNA-320b, with downstream expression of ubiquitin-specific peptidase 37 (USP37) upregulated secondary to IH and USP37 promoting lung cancer progression by mediating the deubiquitination of Cdc10-dependent transcript 1 (CDT1) (98). Public databases and big data platforms have recently provided us with rich research resources. Zhao et al. analyzed 734 patients (230 adenocarcinomas and 504 squamous carcinomas) with NSCLC accompanied by OSA by combining genetic databases. This study confirmed that the metabolic pathway-related genes *HK2* and *GBE1* were significantly altered in lung cancer cells under IH conditions. *HK2* and *GBE1* are located downstream of the HIF-1α signaling pathway, and their expression was positively correlated with tumor size, primary tumor stage T, and tumor lymph node stage N. Meanwhile, higher expression of *HK2* and *GBE1* has also been shown to affect OS in lung adenocarcinoma but not squamous lung cancer (99). From the Gene Expression Omnibus and The Cancer Genome Atlas, Wang et al. used a weighted gene co-expression network analysis screening method to identify four key genes that are shared with OSA and lung cancer, namely modulator of apoptosis 1 (*MOAP1*), chromobox 7 (*CBX7*), platelet-derived growth factor subunit B (*PDGFB*), and mitogen-activated protein kinase 3 (*MAP2K3*). In this study, a model was constructed to predict the prognosis of patients with lung cancer using an innovative machine-learning approach. Although the area under the receiver operating characteristic curve for this model was 0.707, indicating that the model was moderately effective, this study pioneered the combination of artificial intelligence and OSA with lung cancer, providing ideas for future research (100). Qi et al. also applied a machine learning approach to the study of OSA and NSCLC by screening for two OSA-associated target genes (*EXO1* and *KRT6A*) affecting lung adenocarcinoma treatment and prognosis and obtaining three sets of clusters after clustering and analyzing the relevant populations. Patients in Cluster 1 were the most sensitive to conventional chemotherapeutic agents but also had the worst prognosis. Patients in Cluster 2 had a poor response to immunotherapy, which could be attributed to T cell depletion because of the chronic inflammatory response associated with long-term IH. In contrast, those in Cluster 3 could maximize the benefits of treatment with immune checkpoint inhibitors (101). This finding provides a new biomarker for predicting lung adenocarcinoma prognosis, although the model requires external validation.

### 3.7 Summary

**Figure 5:**
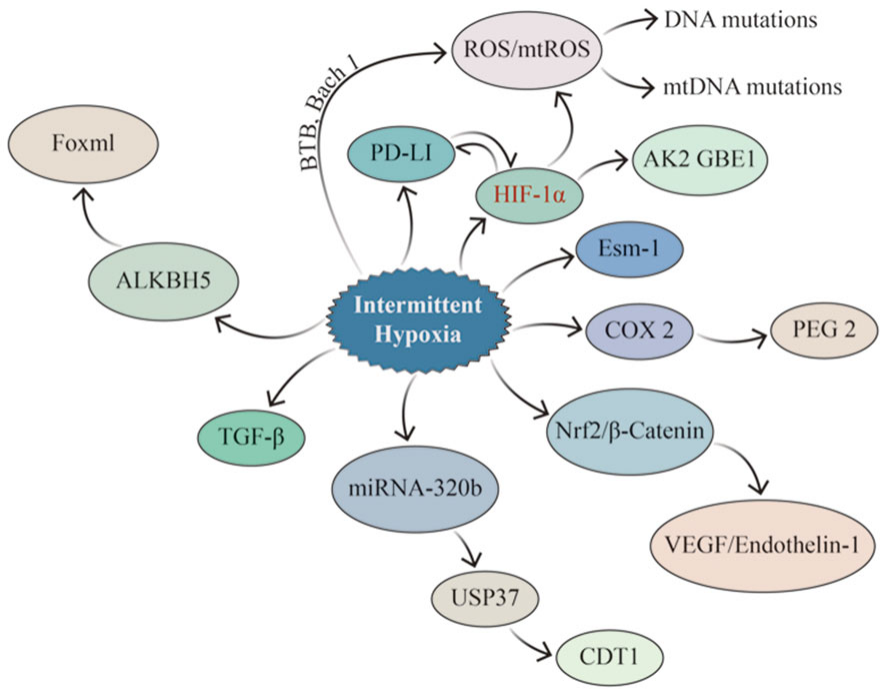
Signaling pathways potentially impacted by IH in lung cancer. IH promotes lung cancer invasion and metastasis by upregulating EMT-related proteins through HIF-1α and ESM1, while HIF-1α enhances the properties of CSCs and increases ROS generation (especially mtROS), resulting in altered genetic material. IH can up-regulate the expression of PD-1 and TGF–β, consequently influencing the tumor immune microenvironment. At the same time, there is a mutual interaction between PD-1 and HIF-1α. IH enhances the expression of Nrf2 and Wnt/β-Catenin-related genes, leading to the up-regulation of their downstream target genes, such as VEGF, which promoted angiogenesis in lung cancer. IH up-regulates the expression of ALKBH5 in lung cancer, which led to the increase of FOXM1 protein level in tumor tissues.

The carcinogenesis and progression of lung cancer involve various factors, including genetic mutations, immunosuppression and evasion, and alterations in the immune microenvironment (**Figure 5**). The two core pathophysiological mechanisms of OSA, IH and SF, lead to secondary oxidative stress, chronic inflammatory response, and dysfunction of the neuro-endocrine and immune functions, closely associated with cancer (102). IH can cause changes in cells, factors, and signaling pathways in the TIME, such as remodeling HIF-a1 and TGF-β signaling pathways, activating TAMs, CAFs, and other cells, and promoting PD-1 expression. Meanwhile, scholars have attempted to establish a bridge connecting the pathophysiological link between OSA and lung cancer and have confirmed that IH, which is associated with OSA, results in the overexpression of some signaling molecules that promote cell proliferation by activating the HIF-1α signaling pathway, which further activates downstream signaling pathways (including inflammation, cell proliferation, and neovascularization). CSCs influence and modify the tumor microenvironment, and vascular endothelial cells differentiate and mature under VEGF stimulation to participate in neovascularization, supplying nutrients required for the growth of lung cancer cells. Although HIF-1α responds differently to IH, sustained hypoxia, and acute hypoxia, IH activates HIF-1α more rapidly than sustained hypoxia (103).

Meanwhile, IH and SF can also promote the recruitment of MDSCs to lung cancer tissues, induce the polarization of TAMs, and inhibit the function of Tregs by regulating the immune system. Exosomes are emerging bioactive factors with multiple biological functions closely related to the progression of lung cancer and therapeutic resistance by transporting nucleic acids and proteins, transmitting biological information, and participating in tumor cell migration and differentiation, immune response, and regulation (104). Long-term exposure to IH induces the activation of proto-oncogenes, and the inactivation of oncogenes, and the body eventually loses the regulation of normal cell proliferation/apoptosis. Many of the above factors combine to contribute to the progression of lung cancer, and these factors may also be promising therapeutic targets.

The entire process of lung cancer progression includes carcinogenesis, tumor tissue proliferation, local invasion, and distant metastasis. The studies mentioned above explain the role played by IH in promoting local invasion and tumor tissue proliferation of lung cancer cells. However, one current limitation is that no study has been able to confirm that IH activates lung carcinogenesis, i.e., that there is a direct causal relationship between IH and lung cancer genesis. In future studies, it is necessary to determine if IH has direct tumorigenicity for lung cancer. Second, a lack of standardized animal models adequately explains the heterogeneity between different studies. In addition, as another important pathophysiological mechanism of OSA, SF may be more complex than IH (105). Unfortunately, to date, only one study has explored the effect of SF on lung cancer (55), and even in that study, SF was not specifically quantified. Finally, IH associated with OSA is also complex, with differences in the duration, frequency, and depth of hypoxia leading to large differences in the degree of hypoxia between patients with a similar AHI. Current *in vivo* animal models or *in vitro* cellular models do not simulate the most realistic state of OSA, which underlies the difficulty of the current work.

## 4 Therapeutic options

It has been demonstrated that tumorigenic expression is upregulated in circulating leukocytes of untreated patients with severe OSA and that the upregulation of the expression of these genes can be reversed after 1 month of CPAP treatment (106). Recently, data from another prospective, multicenter clinical study were published. Untreated severe OSA was associated with a poor prognosis in melanoma (HR 2.96, 95% CI 1.36–6.42), and the HR fell by about half with CPAP treatment (HR 1.66, 95% CI 0.71–3.90) (107).

However, clinical studies exploring the efficacy of CPAP in the field of lung cancer combined with OSA are lacking. Meanwhile, current studies are scarce and limited to basic experiments. Campillo et al. found that IH could promote lung cancer progression by activating the inflammatory pathway (COX-2/PGE2), which in turn induced the proliferation and differentiation of TAMs in a mouse model of OSA+NSCLC. The proliferation of primitive tumor cells was inhibited by administering a nonsteroidal anti-inflammatory drug (celecoxib) that also inhibited the polarization of TAMs (69). IH associated with OSA also induces downstream VEGF overexpression by activating the HIF-1α signaling pathway. VEGF levels in serum and tissues were significantly reduced by endostatin treatment. The effect of endostatin treatment for lung cancer was more significant in the IH group than in the normoxia+NSCLC group (80). ESM1 can also be overexpressed via the HIF-1α signaling pathway, and IH promotes NSCLC invasion and migration and induces NSCLC drug resistance by activating ESM1/HIF-1α (75). After the knockdown of ESM1, the tumor growth- and metastasis-promoting effects of IH were significantly inhibited.

The mechanisms by which IH promotes the progression of lung cancer, including induction of PD-L1 expression and upregulation of VEGF levels, have been demonstrated in basic research. Therefore, we speculate that immune checkpoint inhibitors or anti-VEGF monoclonal antibodies might additionally benefit patients with lung cancer and OSA. However, these therapeutic options should be fully validated in preclinical models before they can be used in clinical studies. We are just taking our first steps in the field of therapeutics for OSA and lung cancer.

## 5 Conclusions and prospects

Although the last decade has witnessed advances in our understanding of the connection between OSA and lung cancer, by summarizing the results of the above clinical and epidemiological data and pathophysiological mechanism studies, it is clear that there are more questions than answers.

Current data from human epidemiological studies indicate that OSA is a relatively common comorbidity in patients with lung cancer; therefore, it seems important to include screening for OSA in the clinical evaluation of patients with a first diagnosis of lung cancer. Concurrently, the incidence of lung cancer appears to be higher in patients with OSA than in the normal population, and future prospective clinical studies with larger sample sizes will be required to demonstrate whether OSA is an independent risk factor for lung cancer. Although there is a lack of evidence that OSA leads to a worse prognosis in lung cancer, it would be arbitrary to conclude at this point that OSA does not increase the risk of mortality from lung cancer, as the current level of evidence in this area of research is very low. Therapeutic research on lung cancer and OSA is sparse and preclinical and is still in its infancy. Regarding the current situation of epidemiologic studies, we believe that, in the future, we need to focus on the following aspects for improvement. First, considering the original purpose of the study of the interrelationship between OSA and lung cancer, it is important to establish a large-sample clinical or population cohort with a long follow-up so that the study can determine the carcinogenicity of OSA. This is important because lung cancer is preventable by blocking risk factors (e.g., smoking cessation) (108). Hypothetically, if we can confirm OSA as an independent risk factor for lung carcinoma and provide early intervention (CPAP treatment) for reversible factors (e.g., chronic IH), this would be important for lung cancer prevention. Second, patients should be evaluated using PSG if possible. While traditional parameters such as AHI, ODI, arousal index, sleep stage, and sleep duration were included, some promising and innovative parameters such as hypoxia burden (109), arousal intensity (110), OR product (111), and cardiopulmonary coupling (112) were included that could help establish a causal relationship between OSA and lung carcinoma. Third, important confounding variables, such as shift work, objective sleep duration, and the inclusion of important concomitant symptoms of OSA, such as excessive daytime sleepiness and insomnia, need to be adequately accounted for in studies that are more specifically stratified.

Moreover, to better understand the impact of OSA on the prognosis of lung cancer, it is recommended that a stratified design be based on the pathologic type, analytic type, and clinical stage of lung cancer. Concurrently, a more meticulous setting of study endpoints, such as recurrence rate, metastasis rate, OS, PFS, and 5-year survival rate, is needed. Finally, there is an urgent need for clinical trials to confirm the role of CPAP therapy in patients with lung cancer with coexisting OSA, as well as to validate the efficacy and safety of immune checkpoint inhibitors and anti-VEGF monoclonal antibodies in this particular group.

**Figure 6:**
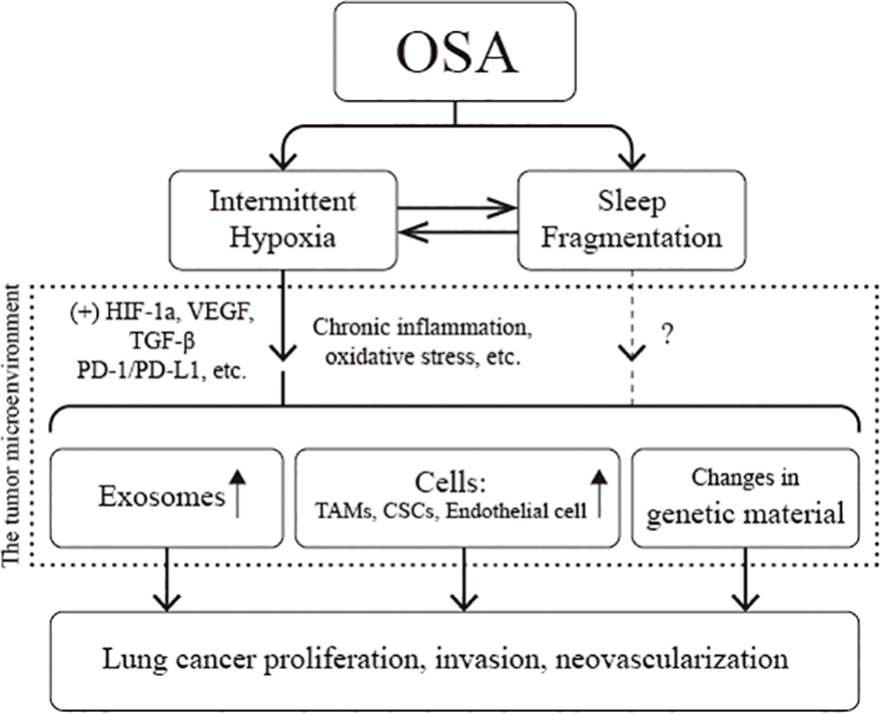
Pathophysiological association of obstructive sleep apnea (OSA) with lung cancer. CSCs, cancer stem cells; HIF-1α, hypoxia-inducible factor-1a; PD-L1, programmed cell death-ligand 1; TAMs, tumor-associated macrophages; VEGF, vascular endothelial growth factor.

Adequate *in vivo* or *in vitro* models provide a more complete and in-depth understanding of lung cancer and OSA association. Current studies have confirmed that IH associated with OSA leads to a secondary inflammatory response, oxidative stress injury, immune dysfunction, and tumor-related gene mutations; upregulates the expression of various signaling molecules, such as PD-L1 and VEGF; and induces the proliferation and differentiation of TAMs, CSCs, and endothelial cells. At the same time, with the help of exosomes, signals regulating cell proliferation and invasion are transmitted between cells. The above multiple pathways promote the proliferation of tumor cells, increase the invasiveness of tumor tissues, induce neovascularization, and ultimately promote the progression of lung cancer (**Figure 6**). The current dilemma is that there is a lack of standardized animal models. Most existing animal models are designed to mimic IH and thus verify the causal relationship between OSA and lung carcinoma. SF, another necessary pathophysiologic process of OSA, is being neglected. Second, the existing *in vitro* or *in vivo* models only demonstrated that IH promotes the proliferation and invasion of lung cancer cells and tissues. However, they did not sufficiently clarify the effect of IH on the distant metastasis of lung cancer and, in particular, failed to validate whether IH can be carcinogenic. Finally, the genesis and progression of lung cancer are related to various factors such as oxidative stress, inflammation, immune evasion, and neuroendocrine disorders; however, a comprehensive study of all these mechanisms is lacking at present. Future studies can consider using EEG to quantify SF and arousal behavior. Utilizing the information from big data platforms and genetic databases, combining genomics, metabolomics, and imaging genomics, and then fully integrating the data through machine learning will help us gain a deeper insight into the pathophysiological mechanisms that interact in OSA and lung carcinoma.

Undoubtedly, the initial stage of the knowledge acquisition process is both a challenge and an opportunity for researchers, and the unknowns in the field provide fertile ground for future research. In the future, through the integration of traditional medicine and artificial intelligence, the multidisciplinary combination of respiratory medicine, sleep medicine, and oncology, and the conjunction of basic and clinical medicine, the pathophysiological mechanism of the association between lung cancer and OSA could be revealed, which would provide us with a theoretical basis for formulating the optimal clinical management strategy.

## Author contributions

FY: Writing – original draft, Writing – review & editing. YH: Writing – original draft, Writing – review & editing. FX: Conceptualization, Investigation, Methodology, Software, Supervision, Writing – original draft, Writing – review & editing. XF: Conceptualization, Investigation, Methodology, Software, Supervision, Writing – original draft, Writing – review & editing.
